# Free Radical Scavenging, Antimicrobial and Immunomodulatory Activities of *Orthosiphon stamineus*

**DOI:** 10.3390/molecules17055385

**Published:** 2012-05-08

**Authors:** Mohammed A. Alshawsh, Mahmood A. Abdulla, Salmah Ismail, Zahra A. Amin, Suhailah W. Qader, Hamid A. Hadi, Nabil S. Harmal

**Affiliations:** 1 Department of Molecular Medicine, Faculty of Medicine, University of Malaya, Kuala Lumpur 50603, Malaysia; E-Mail: zahraa_alnajaar@yahoo.com; 2 Institute of Biological Science (ISB), Faculty of Science, University of Malaya, Kuala Lumpur 50603, Malaysia; E-Mail: salmah_r@yahoo.com; 3 Department of Biological Science, Faculty of Biosciences and Bioengineering, Universiti Teknologi Malaysia, UTM Skudai, Johor 81310, Malaysia; E-Mail: suhaylaqadir@yahoo.com; 4 Department of Chemistry, Faculty of Science, University Malaya, Kuala Lumpur 50603, Malaysia; E-Mail: ahamid@um.edu.my; 5 Department of Microbiology, Faculty of Medicine and Health Sciences, Sana’a University, Sana’a, Yemen; E-Mail: harmal_nabil@yahoo.com

**Keywords:** antibacterial, minimum inhibitory concentration, peripheral blood mononuclear cells (PBMCs), DPPH

## Abstract

*Orthosiphon stamineus* is considered an important traditional folk medicine. In this study ethanol and aqueous extracts of *O. stamineus* were evaluated *in vitro* for their antioxidant, antimicrobial as well as for their immunomodulatory properties on human peripheral blood mononuclear cells (PBMCs). The DPPH radical scavenging method was used for the determination of antioxidant activity, while the antibacterial efficacy was investigated by both disc diffusion method and Minimum Inhibitory Concentration (MIC) against four bacterial strains (Gram-positive and Gram-negative). Furthermore, the immunomodulatory potential of the extracts was investigated through the MTT assay. Aqueous extract of *O. stamineus* exhibited significant free radical scavenging activity with IC_50_ 9.6 µg/mL, whereas the IC_50_ for the ethanol extract was 21.4 µg/mL. The best antimicrobial activity was shown by the aqueous extract of *O. stamineus* against *Staphylococcus aureus*, with inhibition zone of 10.5 mm and MIC value 1.56 mg/mL. Moreover, the results observed from the MTT assay showed that both plant extracts stimulated the PBMCs proliferation *in vitro* in a concentration-dependent manner, but the aqueous extract has remarkable activity against PBMCs. These findings indicate that *O. stamineus* showed high antioxidant activity and may be considered as an immunomodulatory agent.

## 1. Introduction 

Medicinal plants are widely used all over the World as folk medicine for several purposes. They have been used as antibacterial, antioxidant, antiulcer, anti-inflammatory, antiviral, anticancer agents and for the treatment and prevention of different types of illnesses, especially in developing countries where infectious diseases are endemic and health services and hygiene facilities are inadequate. Estimations made by the WHO (World Health Organization) revealed that 80% of people who live in developed countries generally use traditional medicine [1]. Nowadays there has been increasing interest in the investigation of medicinal plants for the discovery of new antimicrobial and antioxidant agents. 

Reactive oxygen species (ROS) and other free radicals are responsible for many diseases, such as arteriosclerosis, heart diseases, aging process and cancer [2]. The free radicals such as ROS, including hydroxyl radicals, superoxide anions, and hydrogen peroxide, play an important role in promoting tissue damage in living organisms. They may lead to cell damage through membrane lipid peroxidation and DNA mutations and as a consequence of that many diseases such as cancer may develop. The antioxidant activity of phenolic compounds was found to be mainly due to their scavenging and redox properties, through neutralizing and quenching free radicals [3]. Nowadays bacterial infections have increased all over the World and antibiotics resistance has emerged as a challenging therapeutic problem, therefore screening of medicinal plants to discover new antibacterial agents has gained priority [4]. An immunomodulator is a substance used for its effect on the immune system or blood cells. Some medicinal plants and their active components such as Ginseng, Echinacea and Astragalus possess immunomodulatory properties and show potential against malignant diseases and infections [5]. Blood mononuclear cells including natural killer (NK) cells have an important function in the defense against bacterial infections, virus-infected cells and malignant cells. High frequency of infections and cancer development are always related to the reduction of NK cell number or cytolytic activity [6]. The most common methods used to investigate the immune-modulation properties of new compounds is the one using the human peripheral blood mononuclear cells (PBMCs) and mice splenocytes as target cells [7,8]. The activation of PBMCs proliferation is always related to the immunomodulating potential of the extract. A few methods are being widely used to evaluate the immunomodulating activity based on the assessment of the proliferation, metabolic activity [such as 3-(4,5-dimethylthiazol2-yl)-2,5-diphenyltetrazolium bromide (MTT) assay] and cell number quantitation (direct cell counting using a hemocytometer) [9].

*Orthosiphon stamineus*, Benth (Family: Lamiaceae), also known as Misai Kucing in Malaysia and Java tea in Indonesia. *O. stamineus* is traditionally used in Southeastern Asia as a herbal tea, diuretic, to treat kidney disorders, for abdominal pain, gout, fever, hypertension, hepatitis, jaundice and diabetes [10,11]. Moreover, it has been scientifically proven that *O. stamineus* exhibits a range of pharmacological properties such as anti-inflammatory, anti-oxidant, anti-bacterial, anti-angiogenesis properties and as hepatoprotective effects [12,13,14,15,16]. *O. stamineus* contains more than 20 phenolic compounds, including nine caffeic acid derivatives, such as rosmarinic acid and 2,3-dicaffeoyltartaric acid, two flavonol glycosides and nine lipophilic flavones [17]. The main components of *O. stamineus* leaves are the polyphenols (caffeic acid derivatives and the polymethoxylated flavonoids) [18]. This study was performed on ethanol and aqueous extracts of *O. stamineus* to determine their antioxidant activity by measuring the DPPH radical scavenging activity, and to investigate the *in vitro* anti-bacterial activity by the disc-diffusion and MIC methods, as well as to assess the stimulation effects against human PBMCs. 

## 2. Results and Discussion

The percentage yields of ethanol and aqueous extracts for *O. stamineus* were found to be 8.1% and 7.6% (w/w) on dry weight basis, respectively.

### 2.1. Antimicrobial Efficacy

The antimicrobial activity results of the investigated extracts are shown in Table 1. It was shown that both extracts of *O. stamineus* exhibited antibacterial activity against Gram-positive bacteria (*Staphylococcus aureus* and *Streptococcus agalactiae*) but not Gram-negative bacteria (*Escherichia coli* and *Klebsilla pneumonia*). 

Among the investigated extracts, the aqueous extract of *O. stamineus* showed significant activity against *Staphylococcus aureus* and *Streptococcus agalactiae*, with inhibition zones 10.5 mm and 8.1 mm, respectively. Moreover, it was found that aqueous extract has minimum inhibitory concentration (MIC) value of 1.56 mg/mL, while the minimum bactericidal concentration (MBC) was 3.13 mg/mL against *Staphylococcus aureus*. On the other hand the same aqueous extract has only moderate activity against *Streptococcus agalactiae*, with a MIC value of 3.13 mg/mL and MBC value of 6.25 mg/mL (Table 2). 

### 2.2. DPPH Activity

*O. stamineus* exhibited high antioxidant activity, as proven by its scavenging activity towards DPPH radicals. There is no significant difference between the IC_50_ of the ethanol extract (21.4 ± 0.104 µg/mL), and the IC_50_ of the synthetic antioxidant compound BHT (21.1 ± 0.031 µg/mL). On the other hand, aqueous extract of *O. stamineus* showed high free radical scavenging activity with IC_50_ (9.6 ± 0.021 µg/mL), which is better than the IC_50_ of the BHT, but still higher than ascorbic acid (4.6 ± 0.006 µg/mL) (Table 3). In addition, the high antioxidant activity of *O. stamineus* which led to more potent radical scavenging effects is certainly associated with the high content of phenolic components. Our previous work reported that the total phenolic contents of *O. stamineus* were 294.3 ± 0.005 mg (gallic acid equivalents) per g of extracts, which was in accordance with the DPPH result [16].

### 2.3. Immunomodulatory Effects on PBMCs

**Figure 1:**
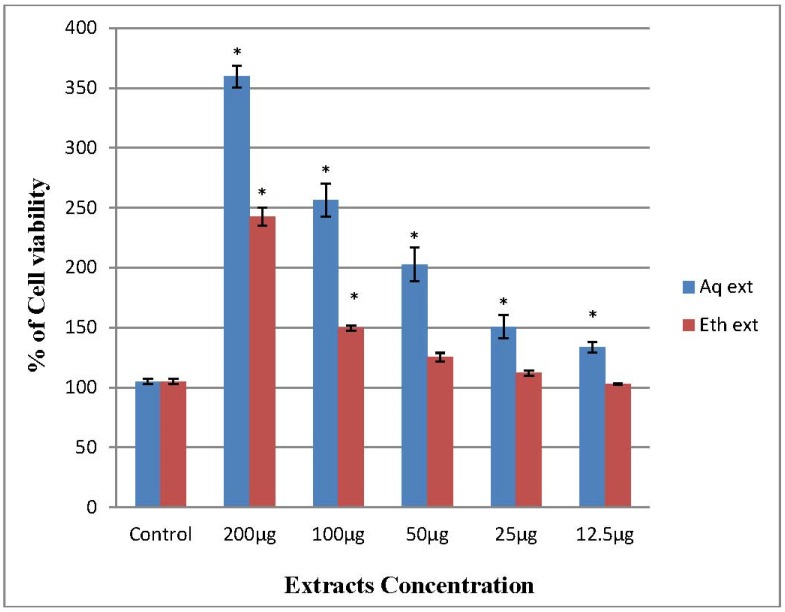
Percentage of (PBMCs) cell viability of *O. stamineus* treated groups compared to control (untreated group).

Both ethanol and aqueous extracts of *O. stamineus* significantly stimulated the proliferation of PBMCs *in vitro* in a dose-dependent manner, but the aqueous extract has remarkable activity against PBMCs. Results showed that the cell viability increased after treatment, it is clear that the extract is not potentially toxic to the immune cells and it is modulating the cellular immune response [8]. Medicinal plants used in folk medicines have been shown to stimulate or suppress immune responses. In our experiment, after an incubation period of 24 h, treatment at 100 µg/mL of aqueous and ethanol extracts of *O. stamineus* significantly increase the number of PBMCs with 256.39 ± 7.98% and 149.75 ± 1.20%, respectively, compared to the control 105 ± 1.30% (Figure 1). On the other hand, there are no significant differences between 50, 25 and 12.5 µg/mL ethanol extract and control. Generally, any immunostimulant agents has the ability to enhance the body’s defense against infections and cancer. Consequently these agents may be used as adjuncts to chemotherapy in immunocompromised patients such as cancer chemotherapy patients to alleviate infections, as well as to remove the residual cancer cells [19]. *O. stamineus* stimulated the PBMCs proliferation to more than double the amount of the initial PBMCs control numbers, which may be of value in combination with other therapies in the treatment of immunodeficiency, cancer, infections and even autoimmune disorders. 

In general, plants are rich in a wide variety of chemical components, such as alkaloids, tannins, flavonoids and terpenoids, which have antimicrobial and antioxidant properties [20]. In *O. stamineus* extracts the antibacterial and free radical scavenging activities reported was related to their high content of rosmarinic acid [15]. Rosmarinic acid and other caffeic acid derivatives have been reported as predominant in *O. stamineus* extracts, which represent 67% of total identified phenolics [17]. A quantitative HPLC analysis for *O. stamineus* ethanol extracts showed that the rosmarinic acid concentration ranged between 0.117 mg/mL and 0.091 mg/mL, depending on ethanol concentration [18].

In another study the antioxidant activity of rosmarinic acid was better than that of Trolox (6-hydroxy-2,5,7,8-tetramethylchroman-2-carboxylic acid) [21]. Moreover, rosmarinic acid was reported to have a number of biological activities *in vitro*, such as anti-carcinogenic, antioxidant, antibacterial, and anti-inflammatory properties [22]. The results of this study are in agreement with the results published by Ho *et al.* [15] who reported the antimicrobial activities of methanol extracts of the *O. stamineus* against selected food-borne bacteria. This plant could represent a wonderful antioxidant agent, which provides prophylaxis against many diseases like arteriosclerosis, heart diseases, and cancers. According to our previous work and Sumaryono *et al.* [16,17] *O. stamineus* contains high content of phenolic compounds and these compounds could affect the immune system due to the hydroxyl groups in their structure. The hydroxyl groups can stimulate the enzyme or electron-transferring system, thus resulting in immunomodulating properties, particularly in proliferation of macrophages and lymphocytes [23].

## 3. Experimental 

### 3.1. Plant Materials and Extraction

*O. stamineus* leaves were obtained from the Ethno Resource Sdn Bhd Selangor Malaysia. The plant was identified and voucher specimen was kept in our laboratory for future reference. A total of 100 g of the dried and fine powdered plant was extracted with 95% ethanol (900 mL) for 48 h, after that the residue was dried and extracted with distilled water by shaking in a water-bath at 70 °C for 4 h. The ethanol and aqueous extracts were filtered through filter paper (Whatman No. 1) and evaporated under low pressure using a Büchi-type rotary evaporator and finally subjected to lyophilization in freeze dryer to give the crude-dried extracts. The dried extracts were stored at −20 °C until used [16]. 

### 3.2. Antimicrobial Assay 

#### 3.2.1. Microorganisms

The following bacteria strains were used as test organisms: *Staphylococcus aureus* (ATCC 25923), *Streptococcus agalactiae* (laboratory isolate supplied from the Clinical Microbiology Laboratory / University of Malaya), *Escherichia coli* (ATCC 25922) and *Klebsilla pneumonia* (ATCC 700603).

#### 3.2.2. Disk Diffusion Method

The antimicrobial susceptibility tests of the isolated organisms were done by the disc diffusion method using the Kirby-Bauer technique [24] and according to the standards of the National Committee for Clinical Technique Laboratory Standards [25]. All tests were performed on Mueller-Hinton agar. The surface of media was uniformly inoculated by bacterial suspension using cotton swab. The bacterial suspension having equivalent turbidity to 0.5 McFarland standards (approximately 1.5 × 10^6^ CFU/mL). Sterile 6.0 mm diameter blank discs (from Oxoid, Hampshire, UK) were used to load 50 µL of the plant extracts (equivalent to 5 mg/disc) dissolved in 5% sterile dimethyl sulfoxide (DMSO) for ethanol extracts and in sterile distilled water for aqueous extracts. The discs were then allowed to dry. Amoxicillin (2 µg/disc), gentamicin (30 µg/disc) and vancomycin (5 µg/disc) obtained from Oxoid Ltd were used as a positive control, whereas solvent loaded discs were the negative control. The extracts, 5% sterile DMSO, sterile distilled water impregnated discs and the standard drug antibiotic discs were placed on Muller-Hinton agar and incubated at 35 °C for 18–20 h. On the next day, the diameters of inhibition zone in mm were recorded. The experiment was done in triplicate and the average diameter of the inhibition zones was calculated.

#### 3.2.3. Minimum Inhibitory Concentration and Minimum Bactericidal Concentration

The Minimum Inhibitory Concentration (MIC) is the lowest concentration of an antimicrobial agent that will inhibit the visible growth of a microorganism after overnight incubation; however, the Minimum Bactericidal Concentration (MBC) is the lowest concentration of an antimicrobial agent where the culture has been completely sterilized after subculture onto antibiotic-free media. The broth micro-dilution method was used to determine the MIC according to the National Committee for Clinical Laboratory Standards guidelines [25]. This assay was done only with the extracts that exhibited considerable activity against bacteria in the disk diffusion method (inhibition zone ≥ 8 mm). Using sterile 96-well plates, each plant extract was subjected to a serial twofold dilution by using sterile nutrient broth. The concentrations of plant extracts were 50, 25, 12.5, 6.25, 3.13, 1.56, and 0.78 mg/mL. Approximately 10 μL of a bacterial cell suspension prepared in nutrient broth, corresponding to 0.5 McFarland standards, was added to all wells except those that served as negative control. Controls for bacterial growth without plant extracts were also included on each plate; plates were then incubated at 37 °C for 18–20 h. The higher dilution of the plant extracts [*i.e.*, lowest concentration] that produced no visible growth of the bacteria (no turbidity) when compared with the control wells was considered as the MIC of the extract. After that, the contents of all wells that showed no visible growth were cultured on nutrient agar and incubated further at 37 °C overnight. The next day, the lowest concentration that showed no single bacterial colony on agar was considered as the MBC. 

### 3.3. Scavenging Activity of DPPH 

The antioxidant activities of the aqueous and ethanol extract were determined using 2,2-diphenyl-1-picrylhydrazyl (DPPH) free radical scavenging assay. DPPH is a molecule that contains a stable free radical. In the presence of an antioxidant, which can donate an electron to DPPH, the purple color will develop due to free DPPH radical scavenging and the change in the absorbance at 515 nm can then be measured. The assay was performed as described by Brand *et al.* [26] with minor modifications. Briefly, 1 mg of ethanol and aqueous extracts was dissolved in 1 mL solvent then diluted to get five different concentrations (25, 12.5, 6.25, 3.125 and 1.56 µg/mL). Ascorbic acid was used as antioxidant standard. A quantity of from each plant extract (5 µL) and standard was mixed with 195 µL of DPPH (40× dilution) in triplicate. The decrease in absorbance value was measured at 515 nm for 2 h with 20 min intervals. The radical scavenging activity was calculated from the following equation and the results were expressed as Mean ± Standard Error (S.E.M.):

[% of radical scavenging activity = (Abs Blank − Abs Sample) /Abs Blank × 100]

### 3.4. Peripheral Blood Mononuclear Cell (PBMCs) Immunomodulatory Assay

#### 3.4.1. PBMCs Isolation and Cell Cultures

Fresh blood was collected from healthy adult volunteers using heparinized tubes. Peripheral blood mononuclear cells (PBMCs) were separated by density-gradient centrifugation over Histopaque 1077 (Sigma-Aldrich, St. Louis, MO, USA). The remnant erythrocytes in the recovered PBMCs layer were eliminated using lysis buffer to lysis the RBCs and washed three times in sterile *phosphate buffered saline* (PBS). The PBMCs were re-suspended in 1 mL RPMI 1640 media (Sigma-Aldrich). The mononuclear cells were counted and adjusted to an appropriate concentration in complete RPMI 1640 supplemented with 2 mM glutamine, 1% penicillin-streptomycin antibiotic (Sigma-Aldrich) and containing 10% (v/v) fetal bovine serum (FBS) (J.R Scientific, Inc, US origin) for further assays [27]. Their viability was determined by trypan blue exclusion test [28] and only cell viability greater than 95%, as assessed by the trypan blue undergoes the (3-(4,5-dimethylthiazol-2-yl)-2,5-diphenyl-tetrazolium bromide, a tetrazole) MTT assay. The cells were then seeded on to 96-well flat bottom sterile tissue culture plates (Jet Biofil, Guangzhou, China) at a density of 5 × 10^4^ cells/mL. All cell cultures were maintained at37 °C in a humidified incubator with 5% CO_2_.

#### 3.4.2. MTT Cell Viability Assay

The effect of the extracts on cell viability of human PBMCs were determined by using the MTT assay as described by Mosmann and Scudiero using MTT reagent (5 mg/mL) [29,30]. Briefly, 100 µL of the isolated PBMCs suspension was cultured in 96-well flat bottom microtiter plates at 5 × 10^4^ cells/mL in RPMI medium containing 10% (v/v) FBS and incubated at 37 °C, 5% CO_2_ and 90% humidity incubator for 48 h. The second day, the cells were treated with 20 µL of two fold serial dilution with final concentration (200, 100, 50, 25 and 12.5 µg/mL) of crude extracts and re-incubated at 37 °C in humidified 5% CO_2_ incubator for 24 h. After 48 h, 10 µL of MTT was added into each well in the plates and again re-incubated for 4 h at 37 °C in humidified 5% CO_2_ incubator. The yellow MTT is reduced to purple formazan in the mitochondria of living cells [29]. Approximately 80 µL of medium with MTT was removed from all wells and 100 µL of dimethyl sulfoxide (DMSO) (Fisher Scientific, Loughborough, UK) was added to each well to dissolve the insoluble purple formazan product into a colored solution. The absorbance of this colored solution measured at 595 nm using power wave x340 ELISA Reader (Bio-Tek Instruments, Winooski, VT, USA). The assay performed in triplicate in three independent experiments. The percentage of cell viability was calculated by the following formula and the results were expressed as mean ± standard error (S.E.M.):

[% Cell viability = (Abs sample − Abs blank) / Abs blank × 100]

### 3.5. Statistical Analysis 

The statistical significance was assessed using one-way analysis of variance (ANOVA) followed by Bonferroni’s multiple comparison test. All values were expressed as mean ± S.E.M., and a value of *p* < 0.05 was considered significant as compared to the respective control group using SPSS programme for windows version 18 (SPSS Inc. Chicago, IL, USA).

## 4. Conclusions

In conclusion, the obtained results indicate that *O. stamineus* exhibits potent antioxidant activity and stimulating activity on human PBMCs which might be useful for therapeutic purposes to prevent ROS disorders and enhance the immune system, and could be used as a potential immunomodulatory agent for tumor immunotherapy. Moreover this plant has some interesting antibacterial efficacy, especially for Gram positive bacteria. In summary, our study demonstrates that the aqueous extract of *O. stamineus* was able to induce the proliferation of immune cells, scavenge dangerous free radicals and inhabit the growth of some bacteria.

## Figures and Tables

Aq ext, aqueous extract; Eth ext, ethanol extract, each value represents the means ± S.E.M. of three independent experiments. Means with * symbol are significantly different *versus* control group, * *p* < 0.05.

**Table 1:** Antimicrobial activity of *O. stamineus* in disk diffusion method.

Plant and Control	Extracts	Extract yield (%)	Average Inhibition Zone Diameter (mm)			
			*S. aureus*	*S. agalactiae*	*E. coli*	*K. pneumonia*
*Orthosiphon stamineus*	Ethanolic	8.1	6.8 ± 0.09	6.5 ± 0.09	__	__
	Aqueous	7.6	10.5 ± 0.20	8.1 ± 0.07	__	__
Amoxicillin 2 µg/disc			NT	13.1 ± 0.15	NT	NT
Gentamicin 30 µg/disc			NT	NT	22.5 ± 0.29	21.5 ± 0.26
Vancomycin 5 µg/disc			13.0 ± 0.12	NT	NT	NT

NT, not tested; ^___^, no inhibition; Inhibition zones including the diameter of the paper disc (6 mm); Values are represented the mean inhibition zone (mm) ± SEM of triplicates.

**Table 2:** Minimum inhibitory concentration (MIC) and Minimum Bactericidal Concentration (MBC) of the investigated extracts.

Plant	Extracts	*S. aureus*		*S. agalactiae*	
		MIC (mg/mL)	MBC (mg/mL)	MIC (mg/mL)	MBC (mg/mL)
*Orthosiphon stamineus*	Ethanolic	NT	NT	NT	NT
	Aqueous	1.56	3.13	3.13	6.25

MIC, Minimum Inhibitory Concentrations; MBC, Minimum Bactericidal Concentrations; BHT, Butylated hydroxytoluene; NT, not tested.

**Table 3:** Free radical scavenging activity (DPPH) of the investigated extracts.

Plant and Control	Extracts	DPPH IC_50_ (μg/mL)
*Orthosiphon stamineus*	Ethanolic	21.4 ± 0.104 ^c^
	Aqueous	9.6 ± 0.021 ^b^
Ascorbic acid		4.6 ± 0.006 ^a^
BHT		21.1 ± 0.031 ^c^

DPPH IC_50_ values represent the mean ± SEM of triplicate experiments. Means followed by the same letters are not significantly different, * *p* < 0.05.
